# The Fabrication of Gelatin–Elastin–Nanocellulose Composite Bioscaffold as a Potential Acellular Skin Substitute

**DOI:** 10.3390/polym15030779

**Published:** 2023-02-03

**Authors:** Ahmad Mus’ab Ahmad Hariza, Mohd Heikal Mohd Yunus, Mh Busra Fauzi, Jaya Kumar Murthy, Yasuhiko Tabata, Yosuke Hiraoka

**Affiliations:** 1 Department of Physiology, Faculty of Medicine, UKM Medical Centre, Jalan Yaacob Latiff, Kuala Lumpur 56000, Malaysia; 2 Centre for Tissue Engineering and Regenerative Medicine, Faculty of Medicine, Universiti Kebangsaan Malaysia, Kuala Lumpur 56000, Malaysia; 3 Laboratory of Biomaterials, Department of Regeneration Science and Engineering, Institute for Life and Medical Science (LiMe), Kyoto University, 53 Kawara-cho Shogoin, Sakyo-Ku, Kyoto 606-8500, Japan; 4 Biomaterial Group, R&D Centre, Nitta Gelatin Inc., 2-22, Futamata, Yao City 581-0024, Japan

**Keywords:** gelatin, elastin, nanocellulose, composite bioscaffold, skin substitute

## Abstract

Gelatin usage in scaffold fabrication is limited due to its lack of enzymatic and thermal resistance, as well as its mechanical weakness. Hence, gelatin requires crosslinking and reinforcement with other materials. This study aimed to fabricate and characterise composite scaffolds composed of gelatin, elastin, and cellulose nanocrystals (CNC) and crosslinked with genipin. The scaffolds were fabricated using the freeze-drying method. The composite scaffolds were composed of different concentrations of CNC, whereas scaffolds made of pure gelatin and a gelatin–elastin mixture served as controls. The physicochemical and mechanical properties of the scaffolds, and their cellular biocompatibility with human dermal fibroblasts (HDF), were evaluated. The composite scaffolds demonstrated higher porosity and swelling capacity and improved enzymatic resistance compared to the controls. Although the group with 0.5% (*w*/*v*) CNC recorded the highest pore size homogeneity, the diameters of most of the pores in the composite scaffolds ranged from 100 to 200 μm, which is sufficient for cell migration. Tensile strength analysis revealed that increasing the CNC concentration reduced the scaffolds’ stiffness. Chemical analyses revealed that despite chemical and structural alterations, both elastin and CNC were integrated into the gelatin scaffold. HDF cultured on the scaffolds expressed collagen type I and α-SMA proteins, indicating the scaffolds’ biocompatibility with HDF. Overall, the addition of elastin and CNC improved the properties of gelatin-based scaffolds. The composite scaffolds are promising candidates for an acellular skin substitute.

## 1. Introduction

The use of biological scaffolds as an acellular skin substitute provides a viable alternative to skin grafts in the management of chronic wounds. A scaffold is an artificial network of natural or synthetic-based constructs made to resemble the extracellular matrix (ECM) of the desired tissue. It acts as a template for tissue regeneration [1] and is typically seeded with cells or supplemented with growth factors, thereby playing the role of cell carriers or an acellular skin substitute [2].

Depending on the intended usage, a scaffold must have certain properties, such as biocompatibility, biodegradability, and alignment with the target tissue’s characteristics and architecture [3,4]. In addition, the fabrication process must be cost effective and feasible for mass production [5]. As such, scaffolds’ properties are highly dependent on the fabrication process and the biomaterials used.

Collagen, the main structural protein in the ECM, is commonly used in the fabrication of scaffolds. As it is relatively easy to procure and has many benefits, most of the available matrix products on the market contain collagen [6]. However, collagen carries the risk of zoonotic transmission [7]. Gelatin is a promising biomaterial that can be used as a substitute for collagen and in developing biological scaffolds [8].

Gelatin is a collagen derivative obtained from the partial hydrolysis of collagen materials upon denaturing the triple helical structure [7]. It comprises the repeating amino acid sequence Gly-X-Y, where X and Y are commonly represented by proline and hydroxyproline, respectively [8]. Like collagen, gelatin contains the arginine-glycine-aspartate (RGD) sequence that binds to integrin proteins, allowing for cell attachment [8]. Unlike collagen, it is much cheaper, and carries no risk of transmitting zoonotic diseases, as it lacks aromatic amino acids such as tryptophan, tyrosine, and phenylalanine [9]. Although gelatin is mainly derived from animals, its denatured property confers it with very low antigenicity, and it produces harmless metabolic products post degradation [8]. Gelatin has been used to coat cell culture plates and construct scaffolds due to its cell binding capacity, biocompatibility, and biodegradability [10]. Despite its diverse benefits, gelatin is hindered by its lack of enzymatic and thermal resistance, as well as its mechanical weakness [8]. To overcome these limitations, gelatin requires either crosslinking or reinforcement with other polymers to form a hybrid or composite [11].

Elastin is the second-most abundant protein found in almost all of the non-rigid tissues in the human body [7]. The major role of elastin is to provide tissues with elasticity and resilience, allowing them to withstand mechanical forces and deformations [12]. Its elastic nature is due to a repetition of the pentapeptide sequence valine-proline-glycine-valine-glycine [13].

Elastin is synthesized by various cells such as fibroblasts, smooth muscle cells, and endothelial cells. Its precursor is tropoelastin, a soluble protein that is characterised by alternating hydrophilic and hydrophobic domains within its structure. Upon secretion by the cells, tropoelastin undergoes extensive covalent crosslinking in the matrix to become insoluble elastin fibres [13]. Although this crosslinking provides elastin protein with the longevity of approximately 70 to 80 years in humans, it also results in poor elastin turnover [12,14]. As adult elastin production is substantially reduced, elastin is poorly restored during wound healing. Although elastin synthesis does occur, elastin fibres produced during healing form a disorganised network that contributes to inadequately elastic scar tissue [13].

As a biomaterial, elastin is available in both soluble and insoluble forms [15]. Soluble elastin can be derived from various animal sources, the most common of which are the bovine neck ligament and porcine aorta [16]. Nevertheless, their use is restricted due to zoonotic disease transmission and religious prohibitions [16]. These issues can be overcome by using alternative sources and improving processing methods [16].

Studies have shown that scaffold fabrication benefits from the inclusion of elastin, particularly in terms of mechanical improvement. Elastin reduced the tensile modulus of collagen-based scaffolds [17], different collagen-polycaprolactone nanofibres [18,19], and chitosan membranes [20]. Likewise, elastin allows for faster and more efficient mesh remodelling and functional tissue formation [21]. Scaffolds made from a collagen–gelatin–elastin composite demonstrated good physicochemical properties and good biocompatibility with dermal fibroblasts [22].

Nanocellulose is simply defined as cellulose in the form of nanostructures, with at least one dimension not exceeding 100 nm [23,24]. Its appealing qualities include a high aspect ratio, low surface area, excellent stiffness and tensile strength, low density, and good biocompatibility [25]. Nanocellulose is cost effective given its abundance, and is completely renewable compared to synthetic materials [26].

Nanocellulose is derived from cellulose, a polysaccharide composed of repeating monomer units of β-d-glucose linked together by β-1, 4-glycosidic linkages [27]. Within the monomer units are three reactive hydroxyl groups that interact with the oxygen atoms of the pyranose ring and the glycosidic linkages, by way of intermolecular and intramolecular hydrogen bonds. These hydrogen bonds stabilise cellulose molecules and allow them to be functionally active [28].

Cellulose fibres are formed from bundles of microfibrils, aggregated from many cellulose molecules by hydrogen bonds. The cellulose fibrils contain both an ordered, or crystalline domain, and a disordered, amorphous domain [24]. Cellulose nanocrystals (CNC), a type of nanocellulose, are isolated by hydrolysing the amorphous domain of the fibrils, leaving behind the crystalline domains that make up the CNC [29].

Despite its benefits, nanocellulose has several drawbacks. It is unbiodegradable in humans, and thus may occupy spaces intended for newly regenerated tissues [23]. The retention of non-degradable materials in the skin is associated with a risk of scar formation [23]. Furthermore, inadequate processing can also increase the risk of an immunogenic response, as certain bacterial, wood-based, and algal nanocellulose may contain endotoxins and β-1, 3-d-glucan that are immunogenic [23].

CNC has been used to reinforce various types of scaffolds. CNC improved the mechanical and fluid absorption properties of chitosan-based composites [30,31], and increased the tensile strength of gelatin–alginate scaffolds [32]. CNC has also been used to create highly porous bilayered collagen scaffolds with improved enzymatic resistance and mechanical strength [33].

Gelatin-based composites have been extensively researched [11], and there are gelatin-based medical devices on the market, such as Gelfoam^®^ and Surgifoam^®^. Although both are mainly used as haemostatics [34], they can also be employed to reconstruct tissues. Gelfoam^®^ has been utilised as a scaffold for bone tissue regeneration [35] and as a carrier for adipose tissue-derived stem cells [36]. Meanwhile, Surgifoam^®^ has been shown to support the chondrogenic differentiation of human adipose-derived adult stem cells [37]. However, the use of these products may be restricted in certain populations due to religious prohibitions, given their porcine sources.

Nitta-Gelatin, a halal-grade gelatin product obtained from buffalo bones, has been previously strengthened with different cross-linkers to evaluate its potential as an acellular skin substitute. Its properties can be modified based on the types and concentrations of the cross-linkers used, while retaining compatibility with skin fibroblasts [38]. Nitta-Gelatin was also combined with collagen and elastin to form composite scaffolds acting as acellular templates for skin regeneration [22]. Additionally, it has been combined with elastin to form hydrogels for cutaneous wound treatment [39].

To date, no scientific data have explored the effects of incorporating elastin and CNC onto a Nitta-Gelatin scaffold. This study was conducted to fabricate and characterise a composite scaffold consisting of gelatin, elastin, and CNC that was crosslinked with genipin. To that end, the microstructure, physical and mechanical properties of the composite scaffolds were assessed. Chemical characterisation of the composite scaffolds was also conducted. Finally, the compatibility of the composite scaffolds with skin fibroblasts was evaluated.

## 2. Materials and Methods

This research was approved by Universiti Kebangsaan Malaysia (UKM) Research Ethics Committee (JEP-2019-688) under FF2019-538. All the studies were performed in controlled facilities under the ISO9001:2015 management system.

### 2.1. Materials

Buffalo gelatin was obtained from Nitta-Gelatin Ltd. (Osaka, Japan) in the form of a high-grade quality powder of gelatin 250 bloom. Water-soluble elastin powder was obtained from broiler skin using a treatment previously described by Kamaruzaman et al. [16]. CNC powder was extracted from an oil palm empty fruit bunch by the Faculty of Science and Technology, Universiti Kebangsaan Malaysia. This extraction method has been previously described in detail [33,40]. Genipin powder was obtained from Wako Fujifilm, Japan. The genipin solution was prepared by dissolving genipin powder in 70% ethanol to form a concentrated solution. The solution was then further diluted in phosphate-buffered saline (PBS; Gibco, Waltham, MA, USA) to obtain a final genipin concentration of 0.1% (*w*/*v*).

### 2.2. Preparation of the Composite Scaffold

A stock solution containing 5% (*w*/*v*) gelatin was first prepared by dissolving Nitta-Gelatin powder in distilled water with constant stirring at a temperature of 40 °C. The stock solution was then mixed until all the powders had been dissolved. Elastin was added to the mixture at a concentration of 0.2% (*w*/*v*). Upon dissolving the elastin powder, different concentrations of CNC were added into separate gelatin–elastin mixtures. The solutions were allowed to stir until a uniform suspension of CNC was achieved. The solutions were then pipetted into a specific mould and frozen at −80 °C for 6 h. The frozen samples were freeze-dried (Ilshin, Dongducheonsi, Republic of Korea) for 24 to 48 h until fully dried, followed by crosslinking with genipin through immersion in a 0.1% (*w*/*v*) genipin solution for 6 h. The samples were subsequently washed three times with PBS before undergoing another lyophilization process to obtain the final crosslinked scaffolds. The composite scaffolds containing CNC concentrations of 0.1% (*w*/*v*), 0.5% (*w*/*v*), and 1.0% (*w*/*v*) were labelled GelE_Nc0.1, GelE_Nc0.5, and GelE_1.0, respectively. Scaffolds containing only gelatin and a gelatin–elastin mixture were designated as the experimental controls and labelled Gel and GelE, respectively. The scaffolds were incised accordingly to perform the physicochemical, mechanical, and compatibility tests. The gross appearance of the scaffolds was also assessed. For cell culture, the scaffolds were sterilised beforehand by immersion in a 70% ethanol solution for 20 min and washed three times with sterile PBS.

### 2.3. Microporous Structure Study

Scanning electron microscopy (SEM), operated at 15 kV, was used to examine the scaffolds’ cross-sectional microstructure. Image J software (Version 1.8; National Institute of Health, Bethesda, MD, USA) was used to measure the pore sizes within the scaffolds. The scaffolds’ porosity was evaluated using the liquid displacement method. The dimensions of the scaffolds were measured using a Vernier calliper. The weight difference of the scaffolds between pre- and post-submersion in absolute ethanol for 24 h was also recorded. All the experiments were performed in triplicates and the porosity was calculated using the following formula:

(1) $$\mathrm{Porosity}=\frac{\mathrm{Wf}-\mathrm{Wi}}{\rho V}\times 100$$

where Wf is the final weight, Wi is the initial weight, ρ is the density of absolute ethanol, and V is the volume of the scaffolds.

### 2.4. Swelling Ratio Analysis

The swelling procedure was performed by immersing the scaffolds in PBS at room temperature for 2 h. Before immersion, the scaffolds were weighed to obtain their dry weight. The swollen scaffolds after immersion were then weighed again. All the experiments were performed in triplicates. The swelling ratio was determined using the following formula:

(2) $$\mathrm{Swelling}\;\mathrm{ratio}=\frac{\mathrm{Ws}-\mathrm{Wd}}{\mathrm{Wd}}\times 100$$

where Ws is the swollen weight, and Wd is the dry weight.

### 2.5. Enzymatic Biodegradation

The biodegradation of the scaffolds was assessed by incubating the samples in 0.0006% (*w*/*v*) collagenase type I (Sigma-Aldrich, St. Louis, MO, USA) prepared in PBS. The samples’ initial weight was recorded, and the samples were incubated in the collagenase solution at 37 °C for 6 h. The samples were then washed with PBS and freeze-dried for 24 to 48 h to obtain the final weight. All the experiments were performed in triplicate. The biodegradation rate was evaluated based on the rate of weight loss per hour:

(3) $$\mathrm{Biodegradation}\;\mathrm{rate}\;\left(\frac{\mathrm{mg}}{\mathrm{hour}}\right)=\frac{\mathrm{WI}-\mathrm{WF}}{t}$$

where WI is the initial weight, WF is the final weight, and t is time.

### 2.6. Energy Dispersive X-ray

The elemental composition on the surface of the scaffolds was analysed via energy dispersive X-ray spectrometry (EDX) conducted using a Phenom ProX SEM microscope (Phenom, Eindhoven, The Netherlands).

### 2.7. Fourier-Transform Infrared Spectroscopy

Chemical characterisation of the scaffolds was performed using Fourier transform infrared spectroscopy (FTIR). A small portion of the scaffolds were analysed, and the spectral data were recorded using a Spectrum 400 FTIR spectrometer (Perkin Elmer, Waltham, MA, USA). The measurement was performed at 400 to 650 cm^−1^, at a resolution of 2 cm^−1^ per point, at room temperature. The absorbance peaks were analysed to identify the chemical structure and changes resulting from the fabrication of the composite scaffolds.

### 2.8. X-ray Diffraction Study

The x-ray diffraction (XRD) analysis of the scaffolds was performed using radiation at room temperature in the −2 scan mode. The diffraction patterns were captured using an XRD diffractometer model, D8 Advance (Bruker, Billerica, MA, USA), using CuKα radiation (λ = 1.542 Å) at 35 kV and 10 mA. The samples were scanned with 2θ (where θ represents the Bragg angle) varying from 10° to 70° in a continuous mode. The results obtained were analysed using integrated software to identify the specific peaks.

### 2.9. Thermal Stability Study

Thermal stability evaluation was performed via thermogravimetric analysis (TGA) for the measurement of the samples’ mass change as a function of temperature in a controlled environment. The thermal stability of the samples was measured with a simultaneous thermal analyser (STA), model STA 449 F3 Jupiter (NETZSCH, Burlington, MA, USA). All tests were conducted in a nitrogen-containing environment at a heating rate of 10 °C/min between 50 °C and 800 °C.

### 2.10. Mechanical Evaluation

The mechanical properties of the scaffolds were evaluated using an Instron 8874 linear fatigue testing system (Instron, Norwood, MA, USA). The samples were cut into a rectangular shape measuring 1 cm by 3 cm, and their thickness was measured at three different points on each sample to obtain the average thickness. The samples were then attached to the instrument using a clamp on both ends. A 50 N load transducer at a crosshead velocity of 0.05 mm/min was used to evaluate the mechanical strength. The tensile strain and Young’s modulus of the scaffolds were recorded. All the experiments were performed in triplicate.

### 2.11. Skin Samples

Redundant skin samples were obtained from all consenting healthy patients undergoing abdominoplasty, face lift surgery, or circumcision at Hospital Tuanku Muhriz UKM and KPJ Ampang Puteri Specialist Hospital, with no specificity regarding gender or age groups. All the patients were recruited by convenient sampling. The inclusion criteria include any intact skin from the aforementioned procedures, patients with no known communicable diseases, and skin with no infections. Patients with infectious diseases or any infected skin conditions were excluded from the study.

### 2.12. Human Skin Cell Isolation and Culture

Skin samples approximately 3 cm^2^ in size were cleaned and minced into smaller pieces. The skin was then digested with 0.6% collagenase type I for 5–6 h in an incubator shaker at 37 °C, followed by cell dissociation using 0.05% Trypsin-EDTA (Elabscience, Houston, TX, USA) for 10 min. The dissociated human dermal fibroblasts (HDF) were resuspended in Dulbecco’s Modified Eagle Medium (DMEM; Elabscience, Houston, TX, USA) supplemented with 10% foetal bovine serum (FBS; TICO Europe, Amstelveen, The Netherlands), and the cells were then seeded into three wells (with a surface area of 9.6 cm^2^/well) of a 6-well culture plate. The cells were grown in an incubator at 37 °C in a humidified 5% CO_2_ atmosphere. The culture medium was replaced every two to three days. When the cells reached 70–80% confluency, trypsinisation was performed using 0.05% trypsin-EDTA for 3 to 5 min to dissociate the HDF from the culture surface. Cells in passage 3 were used for the experiment.

### 2.13. Immunocytochemistry Analysis

Cells seeded on the scaffolds were allowed to grow for 24 h followed by washing with PBS, and were subsequently fixed with 4% paraformaldehyde for 10 min. The fixed cells were then permeabilised for 20 min using 0.5% Triton X-100 (Scharlau, Barcelona, Spain) before blocking by immersion in 10% goat serum (Elabscience, Houston, TX, USA) for 1 h at 37 °C. HDF was incubated with the primary antibodies, rabbit anti-human collagen I antibody (1:300 dilution; Abcam, Cambridge, MA, USA) and mouse anti-alpha smooth muscle antibody (1:500 dilution; Abcam, Cambridge, MA, USA) overnight at 4 °C, followed by secondary antibody incubation with Alexa Fluor 647 goat anti-rabbit (Abcam) and Alexa Fluor 488 goat anti-mouse antibodies (Abcam), both diluted to 1:1000, for 2 h at 37 °C. The cells were counterstained with DAPI (Thermo Fisher, Waltham, MA, USA) for 15 min to visualise the nuclei. Between each post-fixing step, the samples were washed with PBS containing 0.1% Tween 20 (Sigma-Aldrich, St. Louis, MO, USA). Images were captured using an A1R confocal laser scanning microscope (Nikon, Minato City, Japan).

### 2.14. Statistical Analysis

All data were presented as mean ± standard deviation of the mean. GraphPad Prism (version 8.0; GraphPad Software Inc., San Diego, CA, USA) was used for statistical analysis. A one-way analysis of variance (ANOVA) was applied to compare the means of multiple groups. A *p*-value of less than 0.05 was considered to be statistically significant.

## 3. Results

### 3.1. Physical and Mechanical Characterisation

**Figure 1:**
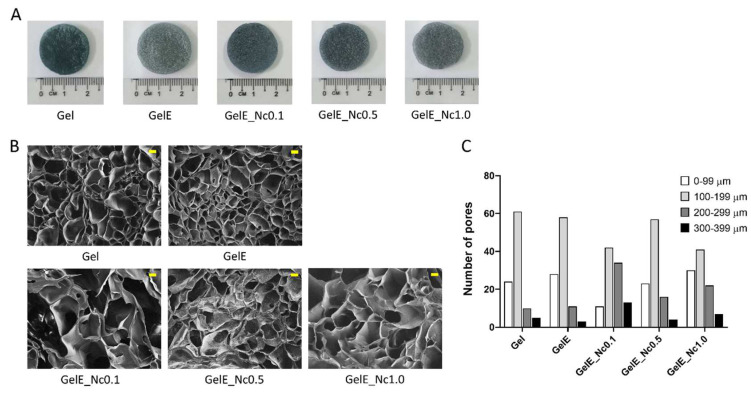
The gross appearance and microstructure of gelatin scaffolds. (**A**) All the scaffolds displayed blue coloration. (**B**) SEM micrograph showing the microporous structure of the scaffolds. Image taken at 50× magnification. The yellow scale bar equals to 100 μm. (**C**) Pore distribution based on the size. Highly uniform pore sizes can be found in the control groups Gel and GelE, and in the composite group GelE_Nc0.5.

Grossly, all scaffolds displayed blue colouration after crosslinking with genipin (Figure 1A). SEM analysis revealed highly interconnected porous structures with thin walls across all scaffolds. The majority of the pores have diameters within the range of 100–199 μm. Based on the pore size distribution, highly uniform porous structures were observed in the control groups, Gel and GelE, as well as the composite group, GelE_Nc0.5. The pores in the GelE_Nc0.1 group were larger, whereas those in the GelE_Nc1.0 group are the least homogenous in diameter (Figure 1B,C).

**Figure 2:**
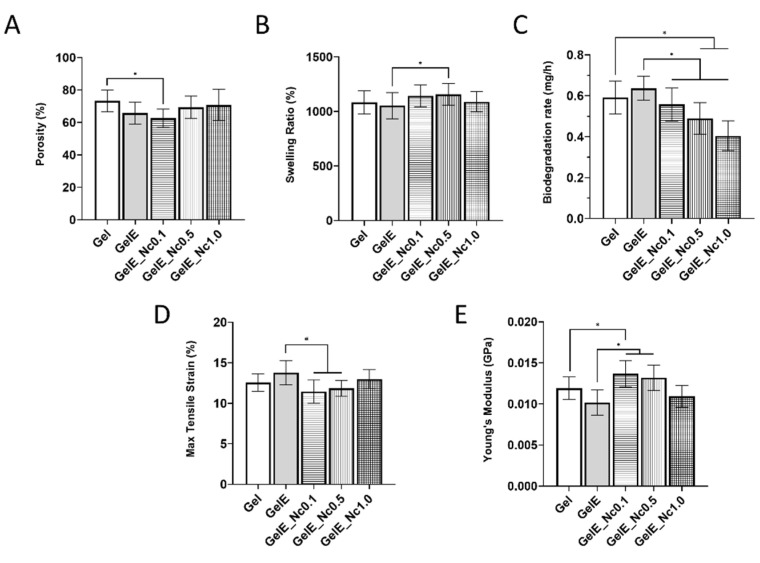
(**A**) Scaffold porosity. When compared to pure gelatin scaffold, the group GelE_Nc0.1 showed significant difference. (**B**) Swelling ratio, indicating the scaffolds’ capacity to absorb fluids. The composite scaffolds displayed a higher swelling capacity compared to the controls. (**C**) Enzymatic biodegradation based on the rate of weight loss per hour. Increased CNC content reduced the weight loss rate of the gelatin scaffolds. (**D**) Maximum tensile strain and (**E**) Young’s modulus of the scaffolds. The control group GelE displayed the lowest stiffness, followed by the composite group GelE_Nc1.0. * represents a significant difference (*p* < 0.05) between groups.

All scaffolds exhibited porosity greater than 60%. The pure gelatin scaffold recorded the highest porosity, at 73.19 ± 6.61%, while the GelE group had a reduced porosity at 65.69 ± 6.79%. The porosity in group GelE_Nc0.1 was significantly reduced to 62.53 ± 5.62%. As the CNC concentration was increased, the porosity also increased to 69.27 ± 6.91% and 70.72 ± 9.61% for GelE_Nc0.5 and GelE_1.0, respectively (Figure 2A).

All scaffolds displayed swelling ratios of more than 1000%. When elastin was added to the gelatin scaffold, the swelling ratio reduced from 1084 ± 105.6% (Gel) to 1053 ± 120.0% (GelE). The presence of CNC slightly improved the swelling capacity, with only GelE_0.5 (1157 ± 99.0%) showing a significant difference from GelE (*p* < 0.05). The swelling ratios of groups GelE_Nc0.1 and GelE_Nc1.0 were 1142 ± 101.2% and 1089 ± 92.8%, respectively (Figure 2B).

The scaffolds’ enzymatic stability was measured as the rate of weight loss for the first 6 h of incubation in collagenase. The biodegradation rate of the control group Gel was 0.592 ± 0.08 mg/h. The GelE group had a slightly higher biodegradation rate of 0.637 ± 0.06 mg/h. With the addition of 0.1% (*w*/*v*) CNC, the biodegradation rate reduced to 0.557 ± 0.08 mg/h. The biodegradation rate decreases as the CNC concentration increases, with the groups GelE_Nc0.5 and GelE_Nc1.0 having rates of 0.490 ± 0.08 mg/h and 0.405 ± 0.07 mg/h, respectively. While the group GelE_Nc0.1 had a significantly lower biodegradation rate compared to GelE only, the latter groups have significantly lower rates compared to both Gel and GelE (Figure 2C).

The mechanical strength of the scaffolds was evaluated based on tensile strain (elongation) (Figure 2D) and Young’s modulus (stiffness) (Figure 2E). In comparison to the pure gelatin scaffold, the addition of elastin reduced the stiffness of the GelE group while increasing the maximum tensile strain. The composite scaffold with the least amount of CNC had the highest tensile strength, with a modulus that was significantly greater than the two control groups (*p* < 0.05). A trend toward reduced stiffness was observed as the CNC concentration increased. While the group GelE_Nc0.5 still had significantly different tensile strain and Young’s modulus compared to GelE (*p* < 0.05), the group with the highest CNC content recorded mechanical properties comparable to those of the controls.

### 3.2. Chemical Characterisation

The elemental contents of the scaffolds as evaluated using EDX spectrometry are shown in Table 1. All scaffolds contained the major elements carbon, oxygen, and nitrogen. All scaffolds contained a significantly higher carbon content compared to the other elements. There was no significant difference in elemental composition between the scaffolds.

**Figure 3:**
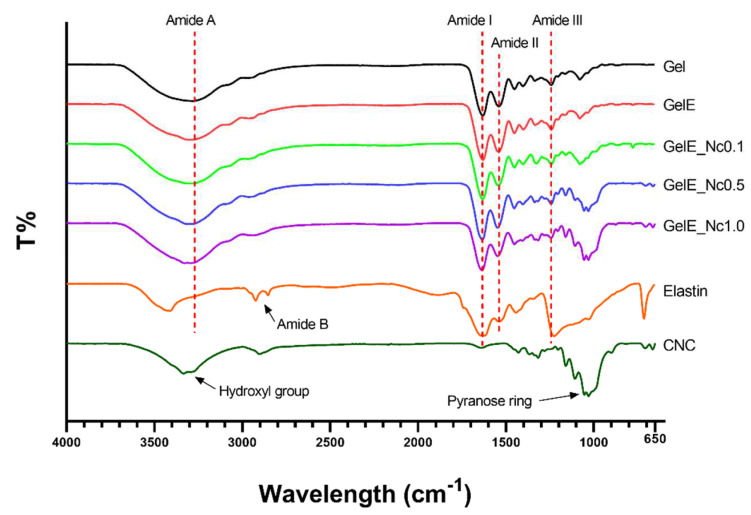
FTIR spectrometry for determining any chemical structure changes for each biomaterial used in the composite scaffolds. Gelatin, the base material, showed no changes in its functional group. Elastin and CNC showed chemical changes when incorporated into the composite scaffolds.

FTIR spectra were obtained for the scaffolds, elastin and CNC powders. All the scaffolds demonstrated characteristic peaks for gelatin at around 3200–3300 cm^−1^ (N-H stretching vibration representing amide A), 1600 cm^−1^ (C=O stretching vibration representing amide I), 1500 cm^−1^ (N-H bending vibration representing amide II), and 1200 cm^−1^ (C-N stretching representing amide III). Elastin powder has major peaks at 3411 cm^−1^, 1622 cm^−1^, and 1537 cm^−1^, which corresponds to amide A, amide I, and amide II, respectively. The amide A peak in elastin appeared to have shifted in the scaffolds, most likely due to its lower concentration compared to gelatin. Elastin powder also has a peak at 2923 cm^−1^ (CH_2_ asymmetrical stretching representing amide B), which was lost in the scaffolds. The CNC-specific peaks were located at 3333 cm^−1^ (O-H stretching vibration) and at 1053 cm^−1^ (C-O-C pyranose ring). The former was not visible in the composite scaffolds, while the absorbance for the pyranose ring became more apparent as the CNC concentration increased (Figure 3).

**Figure 4:**
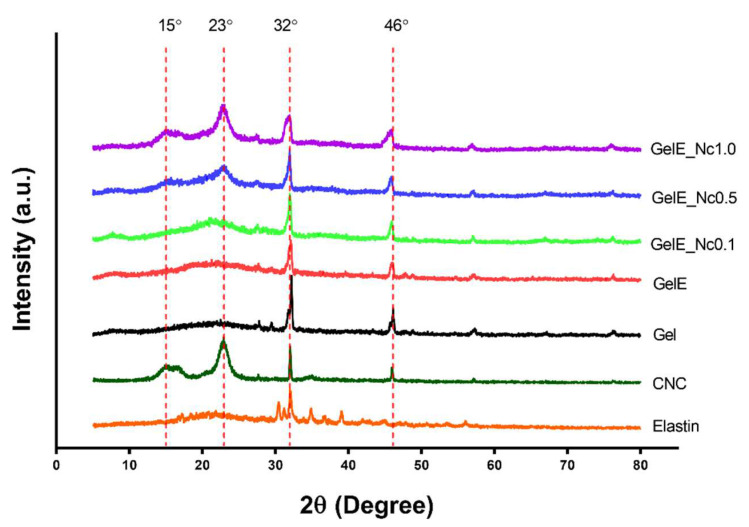
XRD spectrometry for determining changes in the amorphous and crystalline phases for each biomaterial used in the composite scaffold. There are no discernible changes seen for gelatin. Elastin powder displayed similar peaks with gelatin, with minor peaks disappearing in the gelatin scaffolds. Higher CNC concentration increased the peak intensity of CNC within the composite scaffolds.

The crystalline phase present in the scaffolds was examined using XRD spectrometry. The crosslinked pure gelatin scaffold displayed major peaks at 2θ = 32° and 46°, which were preserved across the composite scaffold groups. Elastin powder had several sharp peaks at 30°, 31°, and 32°, the latter of which coincides with the gelatin peak; the former two peaks disappeared in the composite scaffolds, indicating changes in the elastin crystalline structure when mixed with gelatin. CNC had a strong broad peak at 23 °C and a weaker peak at 15°, as well as two sharp peaks similar to gelatin at 32° and 46°. The broad peak at 23° became more prominent with increasing CNC content, and the scaffold with the highest CNC content displayed a broad peak at 15°. Other changes within the composite scaffolds include broadening peaks at 2θ = 32° and 46° (Figure 4).

### 3.3. Thermal Stability

**Figure 5:**
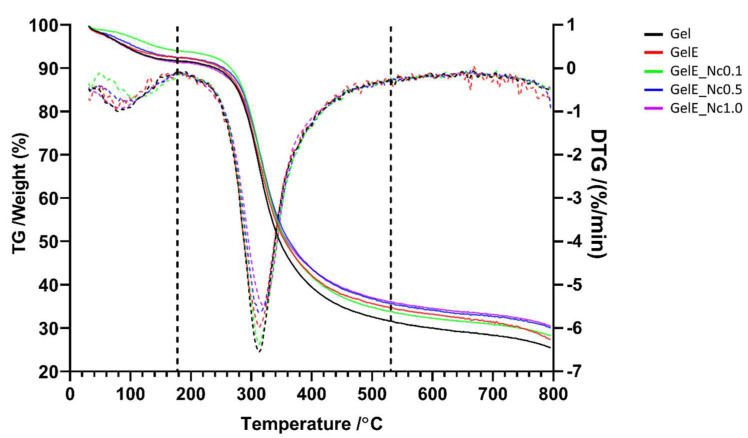
TGA thermogram and DTG curve. Full lines represent the TGA thermogram, showing weight loss for each scaffold as a function of temperature. Dashed lines represent the DTG curve, showing the rate of weight loss for each scaffold as a function of time. Composite scaffolds in general have better thermal resistance compared to the control groups.

The thermal stability of the scaffolds was evaluated with TGA and presented as percentage weight loss in decomposition as a function of temperature. Figure 5 depicts the TGA thermogram and the derivative thermogravimetry (DTG) curve of the scaffolds. 

All the scaffolds exhibited thermal decomposition at three different phases. The first phase occurred between 0 and 180 °C, the second between 200 °C and 530 °C, and the third phase occurred beyond that up to a maximum temperature of 800 °C. The addition of elastin to the gelatin scaffold reduced the percentage of mass loss in the first and second phases, but also lowered the degradation temperature. Despite losing more mass in the third phase, elastin increased the residual mass from 25.4% to 27.1%. When a small percentage of CNC was added to the scaffold, the degradation temperature rises in both the first and second phases, and the residual mass increased slightly to 28.0%. As the concentration of CNC increases, the degradation temperature lowers in the first phase and increases in the second phase. Although increased CNC concentration led to more mass loss in the first phase, it also provided more thermal stability at higher temperatures, with an overall increase in residual mass from 29.9% to 30.4%.

### 3.4. Immunocytochemistry Analysis

**Figure 6:**
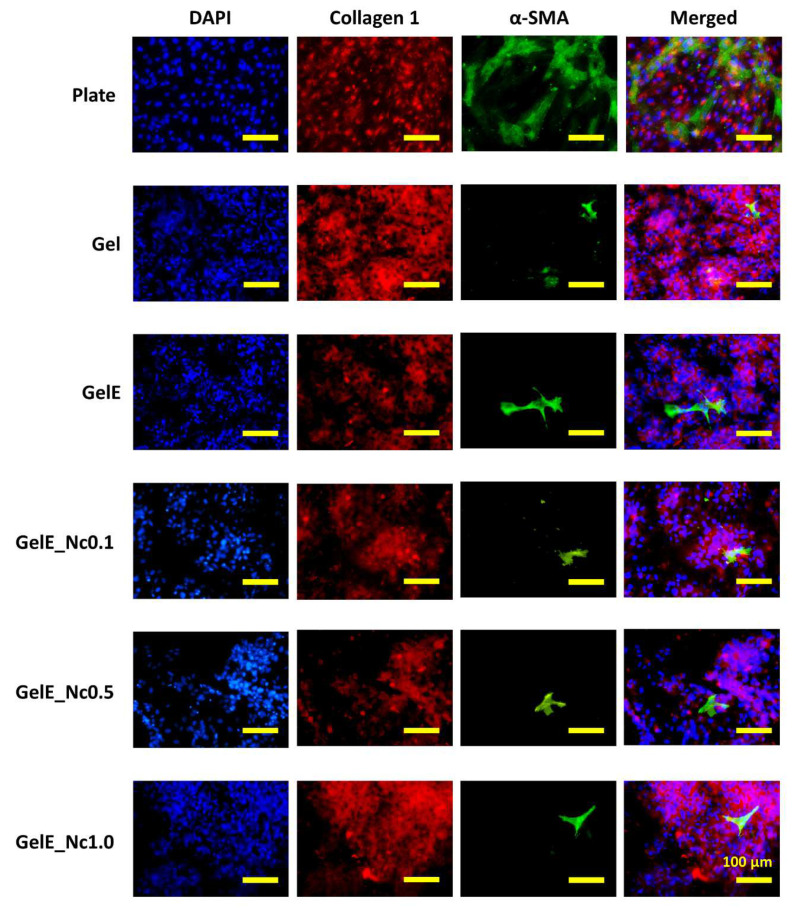
Fluorescent imaging of the HDF cultured on the scaffolds. The group labelled “Plate” represents HDF grown on culture plates. HDF were stained for collagen type I (red), α-SMA (green). Nucleus counterstain was performed with DAPI (blue). All of the proteins were able to be expressed on the scaffolds. The yellow scale bar represents 100 μm.

HDF cultured on the composite scaffolds displayed positive protein expression (Figure 6). Based on the cell population, the HDF were able to attach to the scaffold and proliferate within 24 h after seeding. All of the HDFs on the composite scaffolds expressed good collagen type I protein expression. The expression of α-SMA protein indicated the presence of myofibroblasts on the scaffolds. Compared to the cells on the culture plate (Plate), cells cultured on the scaffolds had lower α-SMA expression. The expression patterns for both proteins on the composite scaffolds were comparable to those of the control groups.

## 4. Discussion

To ensure the effectiveness of biological scaffolds for tissue engineering, their physical and mechanical characteristics must be appropriate for the intended tissue. These characteristics could be tailored accordingly by freeze-drying. Moreover, the properties of scaffolds are influenced by the choice of biomaterials used, and by their cross-linkers. A composite scaffold made from gelatin, reinforced with elastin and CNC and crosslinked with genipin was successfully fabricated for this study using the freeze-drying method.

An important feature when designing scaffolds is their microporous structure. Highly interconnected porous structures with high porosity are required for cell infiltration, proper nutrient diffusion and waste disposal [41]. Although high porosity is often favoured, one must consider the biomaterial itself, as an overly porous structure may adversely affect the scaffolds’ mechanical strength [42] In wound healing, high porosity ensures good oxygen diffusion and moisture retention to enhance the healing process [5]. The optimal porosity for the scaffold is generally accepted to be between 60% and 95% [5,38].

The addition of elastin to the gelatin scaffold reduced its porosity, which may be due to the increase in overall protein concentration [17]. Adding a small concentration of CNC produced the lowest scaffold porosity, while increasing the CNC concentration slightly increased the porosity. A similar trend was observed in a previous study that used the CNC from the same source [33]. In contrast, Shaheen et al. noted that the highest porosity for their chitosan–alginate–hydroxyapatite composite scaffold was achieved with 1% CNC, while increasing the CNC concentration reduced the porosity [31]. The reduction was attributed to the crosslinking action of CNC with the proteins [31].

CNC has no covalent interaction with gelatin unless it is functionalised with aldehyde groups via sodium periodate oxidation [43,44]. CNC could also be modified with diosgenin to allow it to be crosslinked with gelatin via genipin [45]. The primary forces driving the interaction between the charged gelatin and CNC molecules in an aqueous solution are electrostatic charges. The interaction between gelatin and CNC molecules varies depending on the pH of the gelatin solution, as gelatin can exhibit both positively charged amino groups (−NH_3_^+^) in acidic conditions, and negatively charged carboxyl groups (−COO^−^) in alkaline conditions. Due to the sulphate groups (SO_3_^−^) that are bound to the CNC during the extraction process, the suspension of CNC will be constantly negatively charged, regardless of pH [46].

This study revealed that the lowest CNC concentration yields the lowest porosity. It could be posited that the electrostatic interaction between gelatin and CNC works in tandem with the genipin crosslinking reaction by aggregating the molecules together. On the other hand, higher CNC concentrations may cause the formation of large CNC aggregates [32] that are interspersed between the gelatin network, disrupting the genipin crosslinking action.

Besides porosity, pore size must be considered in order to ensure a scaffold’s suitability for the desired tissue. Pore size influences the ligand density on the material surface, which the cells interact with [1]. The pores must be sufficiently large for cells to migrate through, but small enough to establish a highly specific surface area that facilitates efficient binding of a critical number of cells to the scaffold [1]. Relative to the type of cells, small pores may obstruct cell migration and hinder nutrient diffusion and waste removal, thereby forming necrotic regions within the scaffold [42]. Conversely, overly large pores reduce the surface area within the structure, hampering cell adhesion and slowing down cell migration [42].

There is no consensus on determining the ideal pore size for fibroblast proliferation during wound healing. Fibroblasts were determined to have optimal growth in pore sizes ranging from 50 to 160 µm [47]. A later review stated the ideal pore size for adult mammalian skin cell regeneration ranged from 20 to 125 µm [48]. However, the same review also mentioned that increasing the pore size of genipin-crosslinked gelatin hydrogels increased both cell proliferation and ECM secretion, while smaller pores led to cell over-confluence with no ECM deposition [48]. This is supported by studies employing genipin-crosslinked collagen and gelatin scaffolds that recommended pore sizes between 100 and 300 µm for skin fibroblast migration and vascular formation [22,38]. In reality, the optimal pore size may be influenced by the biomaterial itself, given that cell behaviours are governed not only by physical cues from the matrix, but also by the biochemical cues from the scaffold polymer [48].

Pore size is influenced by CNC concentration. Increasing CNC reduced the mean pore size in the chitosan-based composite scaffolds [31], and also in the alginate–gelatin scaffolds [31]. CNC must be uniformly distributed throughout the scaffold, as more CNC causes aggregation and collapse of the scaffold’s inner structure, skewing the findings towards smaller pore sizes [32].

The swelling ratio indicates the scaffold’s capacity to absorb fluids such as physiological buffers and culture medium in vitro, and wound exudates in vivo. This property is influenced by the porous structure and the hydrophilicity of the biomaterial itself. A scaffold’s ability to absorb and retain moisture is crucial, as healing is accelerated in a moist environment [27].

Gelatin is very hydrophilic, able to absorb fluids up to a thousand times its dry weight due to its numerous hydrophilic groups such as –OH, −CONH, and –COOH [49]. The addition of a small concentration of elastin slightly reduced the selling capacity of the gelatin scaffold, which may be due to the denser network formed by the interaction between gelatin and elastin [17]. The possible crosslinking reaction between gelatin and elastin may also reduce the available hydrophilic amino groups [50].

Cellulose is hydrophilic and thus should impart better fluid absorption for the scaffold. The CNC added to gelatin hydrogels increased their swelling capacity. Good CNC dispersion within the hydrogel allowed for the formation of rigid and stable pores. The small dimensions of CNC also provided greater surface area and interstitial volume, which allowed the hydrogel to retain more water [51]. While other studies reported reduced swelling with increased CNC concentration [43,44,45], it should be emphasized that these studies used modified CNC. CNC oxidation with sodium periodate added a dialdehyde group to its structure, which reacts with the free amine group of gelatin through Schiff base formation [43]. Similarly, CNC functionalised with diosgenin could also form crosslinks with gelatin [45]. Increased CNC concentration led to an increase in the degree of crosslinking, which reduces the swelling capacity of scaffolds [38].

Assessment of in vitro biodegradation was conducted using 0.0006% collagenase type I to mimic the skin microenvironment [22]. Scaffolds should be biodegradable, so as not to induce inflammation, while also being stable enough to aid in the regeneration process without needing frequent replacement [33]. At the very least, scaffold degradation must correspond to the target tissue regeneration. For wound healing in general, 3–4 weeks would be ideal [38].

Scaffolds made of gelatin and elastin degrade at a slightly faster rate than pure gelatin. In this study, soluble elastin was used, which should have the best interaction with gelatin [15], and theoretically should enhance the enzymatic resistance. Increased elastin content has been shown to reduce the degradation rate of gelatin scaffolds as protein content increases [50]. Elastin also reduced the weight loss of alginate scaffolds, but the result was statistically insignificant [44]. In contrast, gelatin and elastin hydrogels degraded at a slightly faster rate than pure gelatin hydrogels, which is consistent with the present study [39]. Composite scaffolds made from chitosan and elastin degraded faster compared to pure chitosan scaffolds, which was due to elastin disrupting the crystalline structure of chitosan [20].

This study used soluble elastin obtained from broiler skin by employing a novel method recently described by Kamaruzaman et al. [16]. While their elastin has been shown to have properties comparable to those of commercially available elastin [16], there are admittedly little data elucidating the nature of this particular elastin in scaffold fabrication. It would be prudent to investigate the potential of this elastin as a biomaterial for scaffold fabrication.

The composite scaffolds exhibited more enzymatic resistance compared to the controls. The role of nanocellulose in providing enzymatic stability varied between studies. According to a study of gelatin–bacterial nanocellulose sponges, those with higher levels of bacterial nanocellulose lost more weight. The bacterial cellulose nanofibrils were thought to be thoroughly dispersed within the sponges and interfering with the gelatin crosslinking [52]. Similarly, electrospun gelatin–CNC fibres degraded faster compared to pure gelatin fibres. This observation was attributed to the numerous hydroxyl groups from the CNC that allow for more water absorption, thus catalysing the hydrolytic process [53].

Other studies reported that CNC provided more enzymatic resistance. The high hydroxyl content of CNC and the stability of its β-1, 4-glycosidic linkages against most enzymes provided better resistance for composite gelatin–cellulose nanofibre scaffolds [54]. Additionally, the functionalised CNC acting as cross-linkers increased the scaffold’s resilience against enzymatic biodegradation [44].

The mechanical properties of a scaffold are also influenced by its porosity. Highly porous biomaterials such as chitosan, collagen, and gelatin have extensive empty spaces within their microstructure, which reduces their mechanical strength [42]. Crosslinking with genipin can be used to strengthen collagen and gelatin scaffolds while retaining the desired porosity [22,38].

Both elastin and CNC alter the mechanical properties of the scaffolds investigated in this study. As elastin provides elasticity to tissues, it should have the same effect on fabricated scaffolds. Previous studies have shown that elastin lowers the tensile modulus of scaffolds made from collagen [21], collagen-polycaprolactone [18,19], and chitosan [20]. In contrast, a small amount of CNC increased the tensile modulus significantly. However, the moduli decreased as the CNC content increased, making the scaffolds more compliant. This may be related to the porous structure of the scaffolds. The denser structure with low porosity in the group with the least CNC imparts greater structural strength, rendering scaffolds with high stiffness. Higher CNC content increased the porosity, leading to a lower elastic modulus.

The effect of CNC on the mechanical aspect differs between studies. Increased CNC from OFEFB led to an increase in the Young’s modulus in collagen scaffolds [33]. Likewise, increased CNC obtained from cotton pulp also enhanced the tensile strength of gelatin–alginate scaffolds [32]. In contrast, increased CNC reduced the tensile strength of gelatin films that are indicative of poor adhesion between gelatin molecules and nanocellulose rods [55].

The closest comparable observation can be found in a study conducted by Hivechi et al. using an electrospun gelatin–CNC nanofibres. They noted that the tensile strength and elastic modulus increased as the CNC concentration increased from 0% to 5% (*w*/*w*). The inverse occurred as the CNC content increased further, beyond 15% (*w*/*w*) [53]. Based on other physical assessments, it could be postulated that a small amount of CNC within the gelatin–elastin scaffold formed a highly dense network with low porosity. Increased CNC concentration led to a higher chance of poor dispersion and agglomeration within the scaffold matrix, which then acted as weak points that reduced the tensile strength [53].

We can compare this study with another study by Khalili et al. which used electrospun gelatin scaffolds enhanced with soluble elastin from bovine neck ligament and cellulose acetate for skin regeneration. The tensile strength of their scaffold ranged from 0.4 to 0.7 MPa, compared to the present study in which the modulus was approximately 0.01 GPa (10 MPa). The measurement of the scaffolds’ tensile properties in their wet state, performed in the former study, compared to the dry scaffolds assessed in this study, might explain the differences. Regardless, the results presented in this study are far from recapitulating the natural tensile properties of the skin.

The skin is a very soft organ, with an elastic modulus on the macroscale estimated to be around 60 to 850 kPa. Individual layers have lower moduli, with 35 kPa for the dermis and 2 kPa for the hypodermis [56]. The stiffness of the scaffold matrix is important in that it regulates the cell growth for the desired tissue. For instance, stiffer matrices are more favourable for bone tissue engineering, as they allow for enhanced biomineralisation of the cells and osteoblast adhesion [42].

Ideal biomaterials for skin regeneration should have an elastic modulus below 0.01 GPa [57]. While collagen fibrils have an elastic modulus of around 2–7 GPa, collagen fibres have lower moduli within the 100–360 MPa range. Notably, collagen is responsible for the dynamic mechanics of the skin. While the skin could be deformed with low stress, for example, by pinching, it hardens in response to greater stress due to the reorganisation and stiffening of the collagen network [56].

The present study used a simple mechanical test to evaluate scaffold mechanics on the macroscale. Other techniques, such as atomic force microscopy indentation, may facilitate a more accurate depiction of mechanics on the micro or nanoscale [56]. The mechanics on a smaller scale are better for understanding the mechanical effects of the microenvironment on cell behaviour. Nonetheless, this study highlights the effects of soluble elastin and OPEFB on the mechanics of gelatin scaffolds, which is critical in evaluating their structural stability.

Chemical characterisation is useful for determining whether all the biomaterials are successfully integrated into the scaffold system. The chemical characteristics of genipin-crosslinked gelatin scaffolds were similar to those described by Arif et al. [38]. The functional groups of gelatin and its crystalline structure were preserved in the composite scaffolds.

This study used elastin powder that had been previously characterised by Kamaruzaman et al. [16]. When elastin powder is mixed with gelatin, its amide A peak shifts to a lower wavenumber. This could also be seen when elastin was mixed with chitosan. Increasing elastin content gradually shifted the amide I peak from that of chitosan to that of elastin [20], which can be attributed to alterations in the relative intensity of overlapping bands [58]. As the elastin amide A peak overlaps with that of gelatin and the hydroxyl group of CNC, the shifting could be due to the higher concentration of gelatin and CNC in the scaffold.

There was also a loss of amide B from the elastin powder in the scaffold. While this could be due to the small amount of elastin used, the suppression of native peaks or the formation of new peaks may indicate disruption to the polypeptide chain of elastin during scaffold fabrication [59]. While it could be assumed that this group may be involved in the binding between gelatin and elastin molecules, Khalili et al. stated that a crosslinking reaction between gelatin and elastin is characterised by the formation of a new CH=N bond at 1400 cm^−1^ [50]. Nonetheless, this bond is not discernible from the spectra.

The elemental contents of the composite scaffold groups did not differ significantly according to EDX analysis, which could be attributed to the inhomogeneity of the CNC suspension in the gelatin solution [33]. The XRD peaks of OPEFB CNC powder at 22°, 15°, and 32° are similar to those of CNC isolated from rice husks and pistachio shells and are characteristic of type I cellulose, which indicates successful acid hydrolysis treatment [30,51]. As the concentration of CNC increases, the peak becomes more prominent, indicating increased crystallinity in gelatin scaffolds, thus confirming the CNC content in the composite scaffolds. This could also be seen in another study which used CNC isolated from cotton waste fibres added to gelatin nanofibres. The peak at 22.7° became more prominent as the CNC content of the nanofibres was increased [53].

FTIR spectra showed peaks that are characteristic of CNC, particularly the peaks representing hydroxyl groups and the pyranose ring [33]. As the peak for the hydroxyl group overlaps with the amide A of gelatin and elastin, the peak in this region gradually shifted from that of gelatin to a higher wavenumber as elastin was added and the CNC content increased. Other changes include the increased intensity of the pyranose ring as the CNC concentration increases. Echegaray et al. reported an elevated intensity of the peak at 1048 cm^−1^, which was related to the increasing presence of CNC inside the scaffold [55]. Other minor peaks for CNC powder were located at 2901 cm^−1^, representing C-H stretching vibrations, 1427 cm^−1^, representing C-H bending, and 1315 cm^−1^, representing C-O stretching [30,33,49]. These peaks were also present in the composite scaffold, albeit with slight variations. 

Good thermal stability ensures that the biomaterial can withstand extreme temperatures. Thermal analysis could also be used to determine the effects of the chemical interaction between each of the composite components on the scaffold’s thermal properties.

Gelatin decomposition typically occurs in three steps. From room temperature to around 100 °C, mass loss was caused by the evaporation of water from within the scaffold. The actual decomposition takes place between 100 and 500 °C, where the mass loss is most substantial. Further loss beyond 500 °C is related to the carbonisation of organic compounds [60]. Arif et al. also reported a similar pattern for gelatin decomposition [38].

Elastin addition to the gelatin scaffold only slightly enhanced its thermal resistance, which could be indicative of the possible crosslinking reaction between gelatin and elastin. CNC also slightly improved the scaffold’s decomposition rate. Similar effects of CNC were also observed on the thermal behaviour of gelatin hydrogels [45] and gelatin films [55]. On the other hand, Hivechi et al. noted that CNC caused the gelatin nanofibres to lose more mass in the second phase, which was ascribed to CNC catalysing gelatin degradation [53].

The slight improvement in thermal stability could be due to CNC’s poor thermal stability. Cellulose molecules become thermally unstable after exposure to harsh conditions during nanocellulose extraction [61]. Acid hydrolysis treatment introduced sulphated groups into the crystals, rendering CNC more susceptible to decomposition at high temperatures [62]. Furthermore, the high crystallinity of CNC and the flame retardant behaviour of the sulphate groups have been linked to the high char formation of CNC [30].

Immunocytochemistry was performed to assess the scaffold’s compatibility with HDF by evaluating certain protein expressions. HDFs cultured on the scaffolds expressed collagen type I, indicating that the cells can proliferate and potentially restore the native ECM. It has been demonstrated that collagen-derived proteins stimulate fibroblast proliferation and collagen type I synthesis [63,64].

The expression of α-SMA, although low, indicated the presence of myofibroblasts on the scaffolds. The low myofibroblast population could be due to a lack of regulators that mediate the transition of fibroblasts to myofibroblasts on the scaffolds. The cytokine TGF-β1 is one of the key regulators mediating this transition. TGF-β1 is released from various cells during wound healing such as platelets [65], macrophages [66], and mesenchymal stem cells [67], all of which are absent in in vitro setting of the present study.

Another possible reason is that the gelatin-based scaffolds have low mechanical stress on the microscale. Mechanical stress within the cellular environment also regulates myofibroblast formation. To drive this formation, the culture substrates must be stiff, with an elastic modulus greater than 20 kPa [68]. Ruiz-Zapata et al. depicted a positive correlation between microenvironmental stiffness and α-SMA gene and protein expression in vaginal fibroblasts [69]. Thus, myofibroblast transition is dependent on tissue micro-stiffness, which explains why HDF cultured on the stiffer culture plate expressed more α-SMA than those cultured on the scaffolds.

Tissue stiffness is determined by its protein composition. Elastin is an ECM component that confers elasticity to tissues. A comparison of ECM composition between healthy vaginal tissues and the stiffer pelvic organ prolapse tissue revealed that the prolapsed tissue contained 30% more collagen and 91% more elastin protein compared to the healthy tissue. The prolapsed tissue also had higher levels of α-SMA gene expression [69]. Hence, it can be deduced that increased levels of specific proteins can influence tissue stiffness, thus increasing the myofibroblast population.

Although the aforementioned study documented that collagen and elastin were increased in the stiff prolapsed tissue, collagen may play more role in tissue stiffening. In comparison to elastin, collagen content is 17 times higher in both healthy and prolapsed tissue [69]. Besides, excessive collagen deposition during wound healing is commonly attributed to excessive scarring and skin fibrosis [66]. In the context of tissue stiffening, other ECM components such as collagen may participate more actively in mediating myofibroblast differentiation through tissue stiffness rather than elastin.

Fibronectin is another ECM component that regulates myofibroblast differentiation [68]. HDF cultured on an engineered cleft proliferated rapidly and deposited a large amount of fibronectin on the scaffold surface. Fibronectin increased the surface tension that drives myofibroblast transition to close the gap. It was also found that as the newly formed tissue grows and matures, it lowers the surface tension that reverts the myofibroblasts to the fibroblast phenotype and yields more collagen type I that interlaces with the fibronectin [70]. The transition from fibroblasts to myofibroblasts could be a transient process influenced by tension on the scaffold surface, thereby contributing to the low myofibroblast population on the scaffold.

The main limitation of this study is the lack of a group consisting of solely gelatin and CNC. Unlike the effects of elastin on gelatin scaffolds, there are no similar data for CNC. Thus, the individual effects of CNC on gelatin cannot be compared to those of elastin. Given the narrow scope of this study, only a few physicochemical and mechanical assessments were performed relative to other contemporary studies. Other aspects of wound healing, such as the antioxidant and antibacterial nature of the biomaterials, were also not included in the study. Finally, only one qualitative test was performed to assess the biocompatibility of the composite scaffolds with dermal cells. Thus, the result could only be considered preliminary at this time. Further quantitative analyses would assist in elucidating the effects of the scaffold on HDF behaviour for skin regeneration.

## 5. Conclusions

This study successfully fabricated a composite biological scaffold made from gelatin, elastin, and CNC that was crosslinked with genipin as an acellular skin substitute for skin tissue regeneration. The composite scaffolds demonstrated physical and mechanical properties similar to, if not better than pure gelatin scaffolds. Chemical analysis indicated that while elastin and CNC underwent chemical and structural changes, both biomaterials were incorporated into the gelatin scaffold. A preliminary study based on ICC showed that the composite scaffolds were biocompatible with HDF. More research is needed to investigate the effects of the composite scaffold on skin cell behaviour during wound healing. The use of composite scaffolds could be explored further in a preclinical model to better understand their potential in regenerating skin tissue.

## Figures and Tables

**Table 1:** EDX analysis showing the elemental contents of the scaffolds. All the composite scaffolds displayed similar elemental composition with the control groups.

Groups	Weight (%)		
	Carbon	Nitrogen	Oxygen
Gel	57.29 ± 3.06	22.45 ± 2.15	20.24 ± 2.77
GelE	58.5 3 ± 1.76	24.23 ± 1.31	17.25 ± 2.24
GelE_Nc0.1	61.82 ± 9.19	14.28 ± 4.00	23.90 ± 7.29
GelE_Nc0.5	62.58 ± 4.57	18.79 ± 4.82	18.63 ± 4.38
GelE_Nc1.0	55.46 ± 2.99	21.90 ± 2.83	22.64 ± 2.83

## Data Availability

Not applicable.
